# *N*-Acetylcysteine Reduces Skeletal Muscles Oxidative Stress and Improves Grip Strength in Dysferlin-Deficient Bla/J Mice

**DOI:** 10.3390/ijms21124293

**Published:** 2020-06-16

**Authors:** Paz García-Campos, Ximena Báez-Matus, Carlos Jara-Gutiérrez, Marilyn Paz-Araos, César Astorga, Luis A. Cea, Viviana Rodríguez, Jorge A. Bevilacqua, Pablo Caviedes, Ana M. Cárdenas

**Affiliations:** 1 Centro Interdisciplinario de Neurociencia de Valparaíso (CNIV), Facultad de Ciencias, Universidad de Valparaíso, Valparaíso 2360102, Chile; paz.garcia@postgrado.uv.cl (P.G.-C.); ximena.baez@cinv.cl (X.B.-M.); 2 Centro de Investigaciones Biomédicas (CIB), Facultad de Medicina, Universidad de Valparaíso, Valparaíso 2520000, Chile; carlos.jara@uv.cl (C.J.-G.); marilyn.paz@uv.cl (M.P.-A.); 3 Programa de Farmacología Molecular y Clínica, ICBM, Facultad de Medicina, and Centro de Biotecnología y Bioingeniería (CeBiB), Departamento de Ingeniería Química, Biotecnología y Materiales, Facultad de Ciencias Físicas y Matemáticas, Universidad de Chile, Santiago 8389100, Chile; castorga@fundacioncristovive.cl (C.A.); pablo.caviedes@cicef.cl (P.C.); 4 Instituto de Ciencias Biomédicas, Facultad de Ciencias de la Salud, Universidad Autónoma de Chile, Santiago 8910060, Chile; luis.cea@uautonoma.cl; 5 Escuela Psicología, Facultad de Ciencias Sociales, Universidad de Valparaíso, Valparaíso 2340000, Chile; viviana.rodriguez@uv.cl; 6 Departamento de Neurología y Neurocirugía, Hospital Clínico Universidad de Chile and Departamento de Anatomía y Medicina Legal, Facultad de Medicina, Universidad de Chile, Santiago 8389100, Chile; jbevilac@med.uchile.cl

**Keywords:** dysferlin, dysferlinopathy, oxidative stress, *N*-acetylcysteine

## Abstract

Dysferlinopathy is an autosomal recessive muscular dystrophy resulting from mutations in the dysferlin gene. Absence of dysferlin in the sarcolemma and progressive muscle wasting are hallmarks of this disease. Signs of oxidative stress have been observed in skeletal muscles of dysferlinopathy patients, as well as in dysferlin-deficient mice. However, the contribution of the redox imbalance to this pathology and the efficacy of antioxidant therapy remain unclear. Here, we evaluated the effect of 10 weeks diet supplementation with the antioxidant agent *N*-acetylcysteine (NAC, 1%) on measurements of oxidative damage, antioxidant enzymes, grip strength and body mass in 6 months-old dysferlin-deficient Bla/J mice and wild-type (WT) C57 BL/6 mice. We found that quadriceps and gastrocnemius muscles of Bla/J mice exhibit high levels of lipid peroxidation, protein carbonyls and superoxide dismutase and catalase activities, which were significantly reduced by NAC supplementation. By using the Kondziela’s inverted screen test, we further demonstrated that NAC improved grip strength in dysferlin deficient animals, as compared with non-treated Bla/J mice, without affecting body mass. Together, these results indicate that this antioxidant agent improves skeletal muscle oxidative balance, as well as muscle strength and/or resistance to fatigue in dysferlin-deficient animals.

## 1. Introduction

Oxidative stress (OS) results from an imbalance between the production of reactive oxygen species (ROS) and endogenous antioxidant defenses. This imbalance causes lipid peroxidation and protein oxidation, with the consequent cell dysfunction. In this regard, OS constitutes a mechanism that contributes to the pathology of different conditions, including muscular dystrophies [1,2] and notably dysferlinopathy [3,4,5].

Dysferlinopathies are a group of autosomal recessive muscular dystrophies caused by mutations in the gene encoding dysferlin, a highly expressed skeletal muscle protein critical for sarcolemma repair [6]. The clinical onset of dysferlinopathies commonly occurs between the second and third decade of life, initially impairing lower limb function. Later, paravertebral and proximal upper girdle muscles are compromised, and finally forearm flexor muscles are impaired [7]. The two most common dysferlinopathy phenotypes are Miyoshi’s myopathy and limb girdle muscular dystrophy type 2B [8,9,10]. Muscle biopsies of patients suffering from dysferlinopathies show sarcolemma discontinuities [11], resulting from membrane injury that apparently leads to cytosolic Ca^2+^ overload, with the subsequent mitochondrial dysfunction [12] and ROS production [13]. In this regard, skeletal muscle from dysferlinopathy patients display OS signs, as indicated by increased protein oxidation and lipid peroxidation [3,5,14]. Signs of OS have also been reported in animal models of dysferlinopathy, manifested by increased lipofuscin content, protein thiol oxidation and protein carbonylation [4], and high levels of ROS in isolated myofibers submitted to acute stretch [13,15]. Considering that OS might exacerbate several human pathologies, antioxidant therapies could counteract the progress of dysferlinopathies. In this regard, the co-administration of two antioxidants, resveratrol and coenzyme Q10, reportedly reduced the morphological degenerative and inflammatory features, and improved tissue integrity of skeletal muscle tissue of a dysferlin-deficient SJL/J mice [16,17]. However, a statistical analysis of the effects of these antioxidant agents was not included in such studies.

In the present work, we evaluated the effect of the antioxidant agent *N*-acetylcysteine (NAC) on Bla/J mice, an animal model of dysferlinopathy [18,19]. NAC is an acetylated cysteine residue that can directly scavenge ROS [20], and it has been proposed as a therapeutic option for diseases characterized by oxidative stress [21], including muscular dystrophies such as Duchenne muscular dystrophy [22,23]. In this work, we quantified the effect of NAC on the levels of lipid peroxidation, carbonyls and endogenous antioxidants in quadriceps and gastrocnemius muscles of Bla/J mice. Grip strength was also evaluated. The analyses show that NAC significantly reduced lipid peroxidation and protein carbonyls in the aforementioned muscles, and improved grip strength, suggesting that an antioxidant therapy can ameliorate functional impairment in this type of muscular dystrophy.

## 2. Results

### 2.1. Increased Lipid Peroxidation and Carbonyl Protein in Quadriceps and Gastrocnemius of Dysferlin-Deficient Bla/J Mice Are Ameliorated by NAC Supplementation

**Figure 1:**
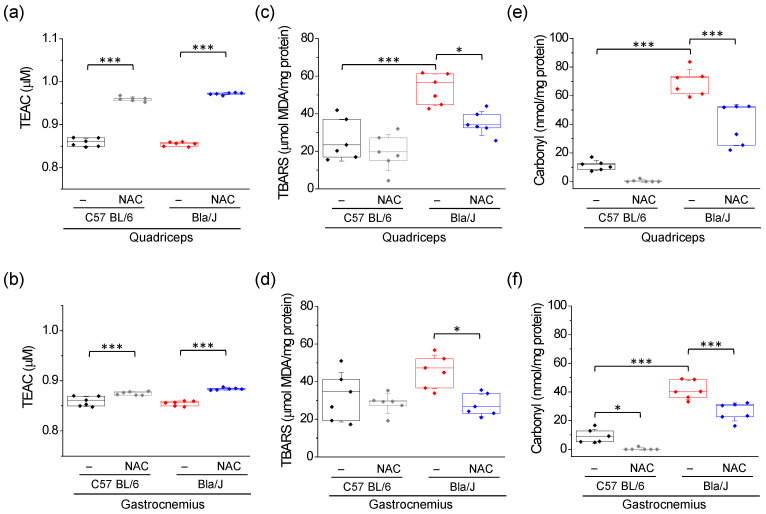
Effects of NAC on Trolox equivalence antioxidant capacity (TEAC), lipid peroxidation and protein carbonyl on quadriceps and gastrocnemius muscles of dysferlin-deficient Bla/J mice. TEAC (**a**,**b**), thiobarbituric acid reactive substances (TBARS), a lipid peroxidation marker (**c**,**d**), and protein carbonyl content (**e**,**f**) were measured in quadriceps (upper panels) and gastrocnemius (lower panels) of C57 BL/6 or Bla/J mice untreated or treated with 1% NAC for 10 weeks. Boxes indicate 25–75 percentiles, lines within boxes indicate medians, and whiskers indicate SD. Each dot represents an individual sample (six mice per group). * *p* < 0.05; *** *p* < 0.001 (one-way ANOVA followed by Tukey-Kramer multiple comparisons test). All data pass the Kolmogorov–Smirnov normality test.

The Bla/J mouse model was developed by backcrossing A/J mice (a naturally occurring dysferlin-deficiency mouse) onto the C57BL/6 background, therefore C57 BL/6 mice serve as control [18]. A/J and Bla/J mice display similar dystrophic characteristics, but Bla/J mice do not exhibit impairments such as poor fertility and susceptibility to infection, which are not observed in dysferlinopathy patients, but are present in A/J mice [24]. Here, we examined the effect of a ten weeks treatment with 1% NAC in 6-months old C57 BL/6 and Bla/J mice, and analyzed total antioxidant capacity (TAC), and lipid and protein oxidation status of quadriceps and gastrocnemius muscles at the end of the treatment. TAC, an index of the total antioxidant strength of a molecule, was measured using the 6-hydroxy-2,5,7,8-tetramethylchroman-2-carboxylic acid (a water-soluble vitamin E analog known as Trolox) equivalent antioxidant capacity (TEAC) assay [25]. This index was not significantly different in quadriceps and gastrocnemius of C57 BL/6 and Bla/J mice (Figure 1a,b). As expected for an antioxidant supplementation [26,27], NAC treatment significantly increased TEAC values in quadriceps and gastrocnemius muscles of both C57 BL/6 and Bla/J mice (Figure 1a,b, and Table S1). Then, these data indicate that NAC effectively increased TAC in muscles from both animals.

We also measured levels of MDA, a marker of lipid peroxidation, and protein carbonylation, which is generated by oxidation of amino acid residues such as proline, arginine, lysine and threonine among others [28]. Compared with C57 BL/6 mice, quadriceps but not gastrocnemius of Bla/J mice had significantly higher levels of lipid peroxidation, as evidenced by MDA contents (Figure 1c,d). Treatment with NAC decreased lipid peroxidation in both muscles of Bla/J mice (Figure 1c,d).

Carbonyl levels were significantly higher in both quadriceps and gastrocnemius of Bla/J mice, and NAC treatment reduced this protein oxidation marker in both muscles, but not to levels of NAC treated C57 BL/6 mice (*p* < 0.001; Figure 1e,f). NAC also significantly reduced carbonyl levels in gastrocnemius of C57 BL/6 mice. Data (means ± SD) of these results are shown in Table S1.

### 2.2. Effects of NAC Supplementation on SOD and Catalase Activity in Quadriceps and Gastrocnemius of Dysferlin-Deficient Bla/J Mice

SOD and catalase constitute an important antioxidant system in the skeletal muscle; SOD catalyzes the transformation of superoxide anion to hydrogen peroxide, and catalase dismutates hydrogen peroxide into water and molecular oxygen. As these enzymes are upregulated in a ROS-dependent manner, their activities can be used as markers of oxidative stress [29].

**Figure 2:**
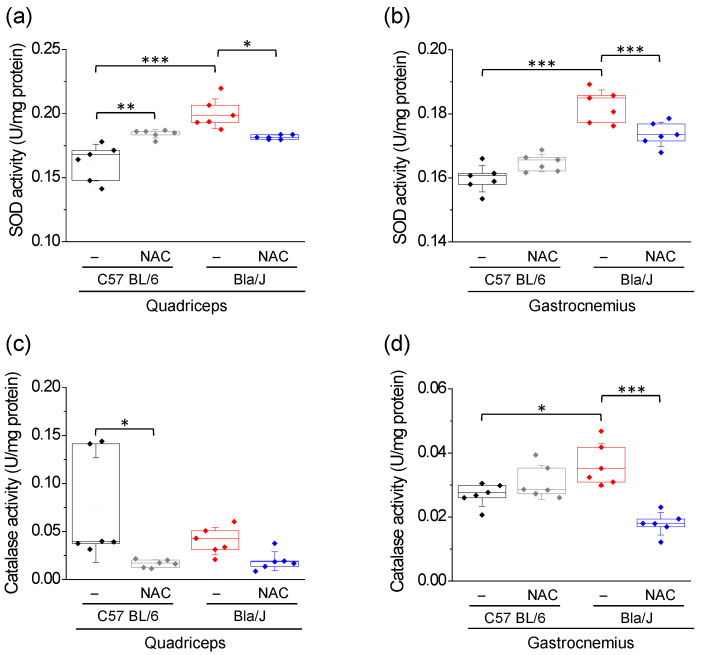
Effects of NAC on SOD and catalase activity in quadriceps and gastrocnemius of dysferlin-deficient Bla/J mice. SOD (**a**,**b**) and catalase (**c**,**d**) activities were measured in quadriceps (left panels) and gastrocnemius (right panels) of C57 BL/6 or Bla/J mice untreated or treated with 1% NAC for 10 weeks. Boxes indicate 25–75 percentiles of SOD or catalase activities, lines within boxes indicate medians, and whiskers indicate SD. Each dot represents an individual sample (six mice per group). * *p* < 0.05; ** *p* < 0.01; *** *p* < 0.001 (one-way ANOVA, followed by Tukey-Kramer multiple comparisons test). All data pass the Kolmogorov–Smirnov normality test.

As shown in Figure 2a,b, SOD activity is significantly increased in both quadriceps and gastrocnemius muscles of Bla/J mice, as compared with those of C57 BL/6 mice (see means ± SD in Table S1). NAC supplementation significantly reduced SOD activity in both muscles, although the activity level of this enzyme in gastrocnemius did not reach that observed in NAC treated C57 BL/6 mice (*p* < 0.001; Figure 2b). NAC treatment also increased SOD activity in quadriceps, but not in gastrocnemius of C57 BL/6 mice (Table S1).

Catalase activity in quadriceps of C57 BL/6 mice displayed a large dispersion and was significantly reduced with NAC supplementation (Figure 2c). Furthermore, the activity of this enzyme in the quadriceps of Bla/J mice was not significantly different from that observed in C57 BL/6 mice, and it was not affected by NAC supplementation (Figure 2c). On the other hand, catalase activity in gastrocnemius of Bla/J mice was significantly elevated as compared to that observed in C57 BL/6 mice and was significantly reduced by NAC supplementation (Figure 2d).

### 2.3. NAC Treatment Has no Effect on Muscle Mass of Dysferlin Deficient Bla/J Mice

**Figure 3:**
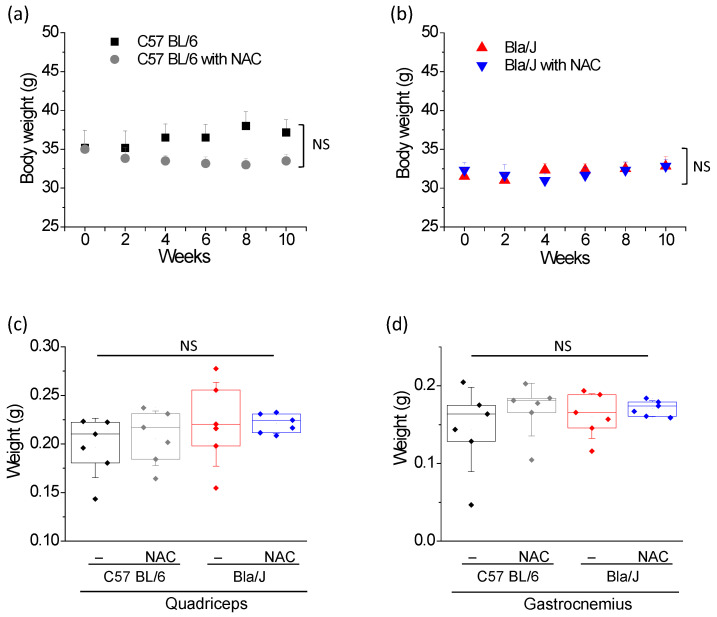
Effects of NAC on and body mass and muscle weights in C57 BL/6 and dysferlin-deficient Bla/J mice. Body mass was controlled immediately prior to the onset of the treatment (week 0) and then every two weeks in C57 BL/6 (**a**) and Bla/J mice (**b**). Quadriceps (**c**) and gastrocnemius (**d**) wet mass were evaluated after finishing NAC supplementation. In panel (**a**,**b**), data show mean ± SE of body weight. No significant (NS) differences were found (paired ANOVA for repeated measures) between body weights of non-treated and NAC treated animals over the 10 weeks of treatment. In panels (**c**,**d**), boxes indicate 25–75 percentiles of muscle weights, lines within boxes indicate medians, and whiskers indicate SD. Each dot represents an individual datum (six mice per group). No significant (NS) differences were found (one-way ANOVA followed by Tukey-Kramer multiple comparisons test). All data pass the Kolmogorov–Smirnov normality test.

Weight loss in muscular dystrophy is generally associated to muscle wasting [30], a condition also reported in dysferlinopathy [6]. Therefore, we monitored the evolution of body mass in control (Figure 3a) and dysferlin-deficient mice (Figure 3b) in parallel to experiments. Immediately prior to the onset of NAC treatment, body mass of Bla/J mice was 31.9 ± 2.3 g (mean ± SD; *n* = 12), being significantly lower (*p* = 0.025, t-test) than that of C57 BL/6 mice (mean ± SD = 35.1 ± 3.9 g, *n* = 12). Although NAC supplementation tended to reduce body mass in C57 BL/6 mice (means ± SD = 37.2 ± 4.0 g and 33.5 ± 2.1 g for non-treated and NAC-treated control mice at the end of treatment), it did not achieve a significant effect (*p* = 0.075, t-test). Paired ANOVA for repeated measures showed no significant difference (*p* = 0.065) between body mass of non-treated and NAC treated C57 BL/6 mice over the 10 weeks of treatment. On the other hand, NAC did not modify body mass of Bla/J mice (means ± SD = 32.8 ± 3.0 and 32. 7± 2.1 g for non-treated and NAC-treated animals).

We also compared wet mass of isolated quadriceps and gastrocnemius muscles (Figure 3c,d). In C57 BL/6 mice with or without NAC supplementation, quadriceps weights were 0.20 ± 0.03 g and 0.21 ± 0.03 g (means ± SD), respectively. In Bla/J mice with or without NAC supplementation, quadriceps weights were 0.22 ± 0.04 g and 0.22 ± 0.01 g, respectively. In the absence of NAC supplementation, gastrocnemius weights were 0.14 ± 0.05 g and 0.16 ± 0.03 g in C57 BL/6 and Bla/J mice, respectively. After NAC supplementation, gastrocnemius weights were 0.17 ± 0.03 g and 0.17 ± 0.01 g in C57 BL/6 and Bla/J mice, respectively. No significant differences were found between wet mass of isolated muscles from C57 BL/6 and Bla/J mice, either with or without NAC supplementation (Figure 3b,c).

### 2.4. Effects of NAC Supplementation on Grip Strength in Dysferlin Deficient Bla/J Mice

Dysferlinopathy, like other muscular dystrophies, progresses with loss of muscle strength in affected patients [31]. Therefore, we evaluated grip strength by using the Kondziela’s inverted screen test [32] immediately prior to the onset of NAC treatment, and at weeks eight and ten of the experiment. Before treatment, latency to fall in C57 BL/6 mice was 101.3 ± 135 s (mean ± SD; *n* = 12 mice) and 33.8 ± 34 s (mean ± SD; *n* = 12 mice) in Bla/J mice, with no significant differences between the two groups (*p* = 0.107, t-test). No significant differences were found between the groups of C57 BL/6 or Bla/J mice assigned to non-treatment or NAC treatment (*p* > 0.05, t-test).

**Figure 4:**
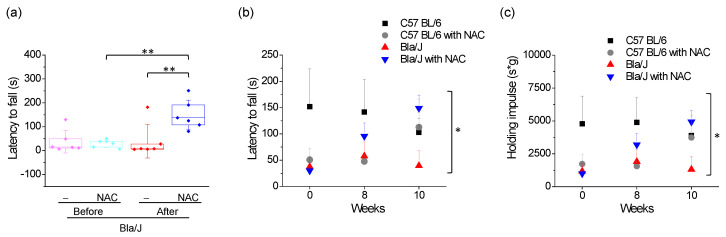
Effect of NAC on grip strength in dysferlin-deficient Bla/J mice. Grip strength was measured using the Kondziela’s inverted screen test. Panel (**a**) Boxes indicate 25-75 percentiles of latency to fall, lines within boxes indicate medians, and whiskers indicate SD. Each dot represents an individual sample (six mice per group). ** *p* < 0.01 (one-way ANOVA followed by Tukey-Kramer multiple comparisons test). Panels (**b**,**c**) compare latency to fall (**b**) and holding impulse (**c**), calculated by multiplying latency to fall by body mass, in the four groups before NAC supplementation (week 0), at week eight, and at the end of treatment (week 10). Data show mean ± SE. Two-way ANOVA with repeated measures revealed a significant interaction between NAC treatment and measurement time in latency to fall (*F*_(1.60, 31.95)_ = 4.53, *p* = 0.025; η^2^ = 0.034) and in holding impulse (*F*_(1.67, 33.39)_ = 3.60, *p* = 0.046, η^2^ = 0.059).

As shown in Figure 4a, NAC significantly improved latency to fall in Bla/J mice as compared with the same group of animals before NAC supplementation, as well as compared with the non-treated group at the end of the experiment (*p* < 0.01; one-way ANOVA followed by Tukey-Kramer multiple comparisons test). Paired t-test between Bla/J animals before and after NAC supplementation yielded a *p* value of 0.0018. Comparison of latency to fall between C57 BL/6 and Bla/J mice without and with NAC supplementation at weeks 0, 8 and 10, is shown in Figure 4b. Values (mean ± SD) of latency to fall are shown in Table S2. Two-way ANOVA with repeated measures revealed a significant interaction between NAC treatment and measurement time (*F*_(1.60, 31.95)_ = 4.53, *p* = 0.025; η^2^ = 0.034). This interaction was not significant in C57 BL/6 mice (*F*_(1.31, 13,11)_ = 1.38, *p* = 0.273, η^2^ = 0.051), but significant in Bla/J mice (*F*_(2, 20)_ = 10.1, *p* = 0.01, η^2^ = 0.129). A post hoc comparison showed a significant difference at week ten in Bla/J mice (t_(15.5)_ = −3.32, *p* = 0.0004).

Figure 4c shows holding impulse (latency to fall normalized by body mass) in the four groups at weeks 0, 8 and 10. Two-way ANOVA with repeated measures revealed a significant interaction between NAC treatment and measurement time (*F*_(1.67, 33.39)_ = 3.60, *p* = 0.046, η^2^ = 0.059). This interaction was not significant in C57 BL/6 mice (*F*_(1.39, 13.88)_ = 0.99, *p* = 0.368, η^2^ = 0.042), but significant in Bla/J mice (*F*_(2, 20)_ = 9.01, *p* = 0.002, η^2^ = 0.121). A post hoc comparison showed a significant difference at the week ten in Bla/J mice (t_(15.8)_ = −3.25, *p* = 0.005).

## 3. Discussion

### 3.1. NAC Restores the Redox Balance in Dysferlin-Deficient Skeletal Muscle

Increased ROS production promotes redox imbalance with the consequent oxidation of biomolecules, such as lipid, protein and nucleic acids, which in turn determine loss of their cellular functions [33]. Thus, OS can lead to altered membrane permeability, disrupted enzymatic activity and DNA lesions, among others [33]. Here, we analyzed the redox status of two skeletal muscles, quadriceps and gastrocnemius, in dysferlin-deficient Bla/J mice, a dysferlinopathy animal model. The first locomotor deficits in Bla/J mice appear at around 15-weeks of age, and morphological alterations and muscle impairment are evident at 4-months of age [18]. The most affected muscles in this dysferlinopathy model are psoas and gluteus, followed by quadriceps and tibial anterior, and then gastrocnemius [18,19]. In mice, both quadriceps and gastrocnemius are mostly composed of type II fibers [34,35], a type of fiber that produces two to three more ROS than type I fibers [36]. Here, we show that both muscles exhibited signs of increased protein oxidation in Bla/J mice. However, quadriceps but not gastrocnemius of Bla/J mice had significantly higher levels of lipid peroxidation, as compared with C57 BL/6 mice. In this regard, the quadriceps seems to be more affected than gastrocnemius in dysferlin-deficient mice. Indeed, quadriceps but not gastrocnemius muscle of 12-months age Bla/J mice exhibited significantly reduced mass, as compared to wild-type animals [19]. Furthermore, in A/J mice quadriceps exhibited higher fat content and protein thiol oxidation than gastrocnemius [4], then suggesting that the first is more susceptible to OS.

Quadriceps and gastrocnemius muscles of Bla/J mice also show altered levels of the antioxidant enzymes SOD and catalase. These two enzymes constitute an important antioxidant system that is upregulated in a ROS-dependent manner in the skeletal muscle [29]. These findings agree with the reported oxidative phenomena (lipid peroxidation and protein oxidation) and altered activities of antioxidant enzymes in dysferlinopathy patients [3,5,14]. High levels of ROS have also been found in skeletal myofibers of dysferlin-deficient A/J mice [13,15], wherein protein carbonylation is observed in over 1-year old A/J mice [4]. As aforementioned, Bla/J and A/J mice exhibit some distinctive features [24,37], among them the amount central nuclei in gastrocnemius [18]. Hence, susceptibility to protein oxidation would be another distinctive feature.

We found that NAC was able to reduce most of the OS signs in quadriceps and gastrocnemius of Bla/J mice, as it reduced lipid peroxidation and protein oxidation in both muscles and tended to restore SOD activity to levels of wild-type muscles. However, NAC also had some effect on C57 BL/6 muscles. It expectedly increased TEAC, as this reflects the antioxidant capability of NAC in muscles, a tissue with a high oxidative activity. On the other hand, NAC changed SOD and catalase activities in quadriceps but not in gastrocnemius of C57 BL/6. The activity of these two enzymes is adapted to the redox status of the tissue [29], and this further depends on the type of muscle fiber [36]. As abovementioned, quadriceps and gastrocnemius are mostly composed of type II fibers in mice. However, quadriceps is almost completely composed of type II fibers [34], whereas gastrocnemius has 16% of fibers type I [35]. This might explain some differences between these two types of muscles.

### 3.2. Effect of NAC Supplementation on Body Mass and Muscle Weight

As loss of muscle weight correlates with muscle wasting in muscular dystrophy [38], we analyzed this parameter in C57 BL/6 and Bla/J mice. Before initiation of NAC treatment, the body masses of Bla/J mice were significantly lower than those of their counterpart C57 BL/6 mice. However, no significant differences were found in quadriceps or gastrocnemius weights from Bla/J and C57 BL/6 mice at the end of treatments (Figure 3). In a previous work, magnetic resonance imaging experiments revealed visibly atrophy in psoas and gluteus muscles from Bla/J mice older than nine months, but no evident changes were observed in gastrocnemius muscles [19]. In the same work was also reported that quadriceps weight of 12-months age Bla/J mice was significantly lower than that of C57B6 mice, but no significant changes were found in gastrocnemius weight [19]. However, quadriceps and gastrocnemius from 4 months old Bla/J mice present centronucleated fibers [18]. Therefore, histological analyses are necessary to determine the progress of the muscular dystrophy in a given muscle.

Regarding the effects of NAC on body mass, no differences were found between this parameter between non-treated and NAC treated Bla/J mice. However, C57 BL/6 tended to lose body mass over the 10 weeks of treatment, although not significantly (*p* = 0.065; paired ANOVA for repeated measures showed no significant difference). Reportedly, NAC supplementation reduced body weights in mice mainly by reducing the amount of visceral fat [39]. The mechanism is seemingly related with its antioxidant properties, as NAC inhibited the increase of body mass caused by a deficiency of the oxidative stress sensor NPGPx [40], as well as by a high fat diet that promotes the expression of genes responsible for lipid oxidation [41]. Supplementation with 2% NAC for six weeks also produced significant loss of body mass, as well as of muscle weight, in the mdx mouse model of Duchenne muscular dystrophy. Nevertheless, this antioxidant agent significantly increased grip strength and muscle maximum specific force [42]. Dystrophic muscles are characterized by inflammation and accumulation of fat and fibrotic tissue [43]. This might explain the effects of NAC on muscle weight in mdx mice, and also the lack of differences in quadriceps and gastrocnemius weights of C57 BL/6 without or with NAC supplementation (Figure 3). In both cases (mdx and Bla/J mice), degenerating muscle fibers may be undergoing replacement with adipocytes and fibrotic tissue, hence compensating for muscle weight loss [43].

### 3.3. Effects of NAC Supplementation on Grip Strength in Dysferlin Deficient Bla/J Mice

Our results using the Kondziela test show no significant differences between C57 BL/6 and Bla/J mice in grip strength before initiating NAC treatment. This is in agreement with the work by Nagy et al. [19], where no difference in grip strength was found in Bla/J mice younger than 67 weeks. Conversely, locomotor activity, measured as total distance travelled, was impaired in 15 weeks age Bla/J mice, as compared with control C57 BL/6 animals. The use of different muscle groups, with different degree of affection in the performance of these types of functional test might explain these differences.

Our analyses also show that NAC supplementation significantly increased grip strength in Bla/J mice as compared with non-treated animals (*F*_(2, 20)_ = 10.1, *p* = 0.01, η^2^ = 0.129; two-way ANOVA with repeated measurements), suggesting that an antioxidant therapy can improve muscle strength and/or resistance to fatigue in dysferlinopathies. The effects of NAC on muscle fatigue have been studied in humans subjected to different types of exercise [44,45]. The mechanism seems to involve increment of glutathione and taurine levels, and reduction of OS [45]. However, we cannot state whether NAC improves muscle strength in dysferlinopathy. Therefore, for a future investigation we propose to evaluate the effects of an antioxidant therapy on in vitro muscle force measurements together with biochemical markers in order to establish more accurately the effects of drugs on dystrophy therapy.

### 3.4. Comparison of the Effect of NAC in Bla/J and mdx Mice

Most of the studies regarding the effect of NAC on muscular dystrophy have been performed in the mdx model of Duchenne muscular dystrophy pathology [22,23,42,46,47], and there are few in other models. One example is an animal model of chronic limb ischemia, where NAC attenuates inflammation [48]. Another example is in a model of peripheral arterial insufficiency, where NAC improves grip strength in soleus but not extensor digitorum longus muscle [49].

**Figure 5:**
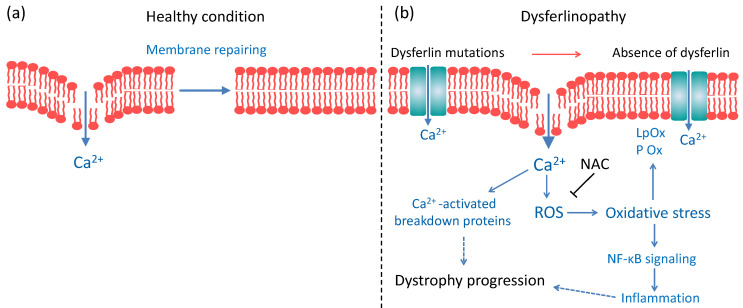
Scheme of mechanisms that might contribute to the dystrophy progression in dysferlinopathies. (**a**) In a healthy condition, the damage in plasma membrane is rapidly repaired, and only a local increase of Ca^2+^ is produced. (**b**) In a dysferlinopathy condition, the membrane repair is impaired [6], and non-selective channels, such as connexin-based hemichannels, P2X_7_ receptors and transient receptor potential TRPV2 channels are *de novo* expressed [56,57]. This could contribute to an altered Ca^2+^ homeostasis [56], mitochondrial dysfunction [12], ROS production [13], and consequently OS, resulting in lipid peroxidation (LpOx) and protein oxidation (P Ox) [4]. OS further conduces to the activation of the NF-κB inflammatory signaling pathway [58]. On the other hand, an altered Ca^2+^ homeostasis might also lead to the activation of breakdown protein pathways such as calpains, ubiquitin-proteasome pathway and autophagy [59], whose overactivation also results muscle atrophy [60]. NAC reduces OS and NF-κB inflammatory signaling pathway [61].

Different muscles are compromised in Bla/J and mdx mice. Studies of respiratory muscle in mdx mice show that the diaphragm is more severe than the observed dystrophy in limb muscles, resembling the disease progression in Duchenne patients [50], whereas Bla/J mice exhibit 50% reduction of force in the diaphragm, compared to control mice [51], but myonecrosis and inflammation in limb muscles increase in response to large strain injury [24]. Studies in mdx mice have shown that treatment with 1% NAC for six weeks prevented ROS production, reduced internal nuclei count and nuclear NF-κB expression [22], as well as myofiber necrosis induced by exercise [23]. Curiously, no effects of such NAC treatment were found on lipid peroxidation and protein carbonylation [23]. On the other hand, a treatment with 2% NAC for six weeks reduced thiol oxidation and inflammation in fast muscles of mdx mice, and improved in vitro muscle contractile function and grip strength [42]. In contrast to the findings reported in mdx mice [23], we found that NAC supplementation for ten weeks was effective in reducing lipid peroxidation and protein carbonylation (Figure 1), suggesting that this antioxidant agent could reverse skeletal muscle OS in dysferlin-deficient mice. These differences in the effects of NAC in these two types of muscular dystrophy might underlie on the molecular mechanism of these diseases. Duchenne muscular dystrophy is caused by mutations in the gene that encodes dystrophin, a protein that forms part of dystrophin glycoprotein complex, which connects the sarcolemma to the extracellular matrix, conferring membrane stability [52]. The malfunctioning of this complex renders the sarcolemma more susceptible to injuries caused by contraction, in turn provoking massive Ca^2+^ entry, mitochondrial Ca^2+^ overload and oxidative stress [53]. The dystrophin glycoprotein complex also includes the nitric oxide synthase (NOS) [54], an enzyme that catalyzes the production of nitric oxide from l-arginine. A dysfunction of NOS also causes OS in Duchenne muscular dystrophy [55]. In the case of dysferlinopathy, as shown in Figure 5, dysferlin deficiency results in impaired membrane repair [6], and de novo expression of non-selective channels, such as connexin-based hemichannels, P2X7 receptors and transient receptor potential TRPV2 channels [56], which might contribute to an altered Ca^2+^ homeostasis, mitochondrial dysfunction [12] and ROS production [13]. This might involve NADPH Oxidase 2 (Nox2)-dependent ROS generation (X-ROS), which is reportedly increased in flexor digitorum brevis muscle of A/J mice submitted to mechanical stretch [15]. Then, only part of the mechanisms that produce OS in Duchenne muscular dystrophy is shared with dysferlinopathies.

### 3.5. Would NAC Be Useful as a Therapy for Dysferlinopathies?

NAC has been proposed as a potential therapeutic agent in a wide spectrum of diseases, such as psychiatric [62], dermatological [63] and pulmonary [64] disorders, as well as in paracetamol overdose [65]. NAC has also been evaluated in individuals with ryanodine receptor 1-related myopathies. However, this antioxidant agent, administered orally for six months, did not reduce OS in these patients [66].

NAC has a strong sulfuric odor and unpleasant taste that reduce the adherence to therapy [67]. The dose of NAC used in this study (around 1.4 mg/g/day) was three times greater than the maintenance oral dose proposed for the treatment of paracetamol overdose [68] and more than 10 times the dose recommended in other illnesses [62]. Furthermore, although NAC successfully improved the redox balance and improved muscle strength and/or resistance to fatigue in Bla/J mice, additional mechanisms may also contribute to the progress of the muscular dystrophy in dysferlinopathy. For instance, the entry of Ca^2+^ through connexin-based hemichannels, P2X7 receptors or TRPV2 channels might also contribute to activation of calpains, whose substrates include cytoskeletal proteins and transcription factors, and their overactivation results in muscle atrophy via ubiquitin-proteasome pathway, and Akt phosphorylation [60]. Knocking-out of connexin 43 and connexin 45 in skeletal muscles prevents the major alterations promoted by dysferlin absence [57]. The absence of dysferlin also promotes altered cytoskeletal actin dynamics [69], which could further lead to an impaired expression of functional protein in the sarcolemma [70]. Therefore, additional therapeutic options should be taken into consideration to address limb-girdle muscular dystrophy, including dysferlin restoration [71].

## 4. Materials and Methods

### 4.1. Animals and NAC Treatments

Bla/J mice (B6.A-Dysfprmd/GeneJ) mice were a kind gift from the Jain Foundation. These animals bear an ETn retrotransposon insertion in intron 4 of the dysferlin gene, leading to the absence of dysferlin protein [37]. Wild-type C57 BL/6 mice were obtained from breeding colonies maintained at the Universidad de Valparaíso (Valparaíso, Chile). The temperature of the animal facility was 21 ± 2 °C.

Genotyping was performed by PCR using DNA extracted from tails. Primers used were: 5′- TTC CTC TCT TGT CGG TCT AG-3′ (forward for C57 BL/6 mice), GCC TTG ATC AGA GTA ACT GTC (forward for Bla/J mice) and 5′- CTT CAC TGG GAA GTA TGT CG -3′ (reverse for both). PCR products were electrophoresed in agarose gels and bands of 207 bp and 234 bp represented C57 BL/6 mice and Bla/J mice, respectively. All animals were housed in 12-h light-dark cycles and received free access to standard mouse chow and water.

NAC (Sigma-Aldrich, St. Louis, MO, USA) was administered as a 1% (*w*/*v*) solution in drinking water ad libitum to 6 months old mice for 10 weeks, according to standard protocols [22,23,42,47]. NAC solutions were prepared every 5 days using autoclaved tap water (pH ~7.0), kept protected from light and refrigerated at 4 °C. Drinker bottles were refilled every two day. As mice drink approximately 5 mL of water per day, we estimated that the NAC dose was approximately 50 mg/day. Considering that the body weight of the mice was in average 35 g per specimen, the daily dose was calculated to be 1.4 mg/g.

This research was approved on 31 March and 29 April of 2016 by the Biosafety and Bioethics committees of Universidad de Valparaíso (Chile), respectively; approval identification numbers BS002/2016 and BEA080-216.

### 4.2. Tissue Collections

At the end of NAC treatment, mice were decapitated under isoflurane (4–5% during anesthesia induction, and 1.5–3% during anesthesia maintenance), and subsequently quadriceps and gastrocnemius muscles were removed. Freshly dissected muscles were weighted and stored at −20 °C, without being previously snap-frozen in liquid nitrogen or dry ice. Later, 100 mg muscle was placed per ml of phosphate-buffered saline (0.15 M NaCl, 0.01 M Na_2_HPO_4_ and 0.01 M NaH_2_PO_4_, pH 7.4) for thawing and then were homogenized using a syringe plunger. Samples were stored again at −20 °C and kept frozen until assay.

### 4.3. Total Antioxidant Capacity (TAC)

TAC, an index of the total antioxidant strength of a molecule, was measured using a water-soluble analog of vitamin E (Trolox) equivalent antioxidant capacity assay. This assay is based in a hydrogen atom transfer that measures the capability of an antioxidant to quench free radicals by hydrogen atom donation [72]. This analysis was carried out in the supernatant obtained after macerating the muscle as previously described by Romay et al. [73]. Briefly, 10 µL of supernatant were mixed with a 1:1 mixture of 150 µM 2,20-azino-bis(3-ethylbenzothiazoline-6-sulfonic acid) (Sigma-Aldrich, St. Louis, MO, USA) and 10 mM 2,20-azobis(2-amidinopropane) (Sigma-Aldrich, St. Louis, MO, USA), previously incubated at 45 °C for 30 min. Radicals presents in the mixture were scavenged by the antioxidants in the sample, and as a result, the colored product concentration decreases over time. Kinetics was determined at 734 nm for 10, 30 and 50 s after adding the sample. The antioxidant capacity thus measured was compared with that of Trolox (Sigma-Aldrich, St. Louis, MO, USA) and expressed in TEAC.

### 4.4. Lipid Peroxidation Measurement

Malondialdehyde (MDA), the main marker in lipid peroxidation, was measured using the thiobarbituric acid reactive substances (TBARS) assay according to Esterbauer et al. [74]. 1 mL of homogenates from quadriceps or gastrocnemius muscles were treated with 30% (*w*/*v*) trichloroacetic acid (TCA; Merck, Darmstadt, Germany) and centrifuged for 15 min at 3000 RPM. Then, 1 mL of the supernatant was mixed with 0.67 % (*w*/*v*) thiobarbituric acid (TBA; Sigma-Aldrich, St. Louis, MO, USA). Samples were boiled for 20 min and their absorbance spectrum was recorded at wavelengths between 400 and 600 nm using a UV–visible Rayleigh UV-2601spectrophotometer (BRAIC Co. Ltd., Beijing, China), and the concentration of the TBA-MDA adduct was determined by extrapolation, from a MDA (Merck Darmstadt, Germany) calibration curve. Each sample was analyzed in triplicate.

### 4.5. Protein Carbonyl Content Assay

This assay is based on the reaction of carbonyl groups generated by protein oxidation with 2-4-ditrophenylyidrazine (Sigma-Aldrich, St. Louis, MO, USA) was performed according to Levine et al. [75]. Ten µL of homogenates from the quadriceps or gastrocnemius muscles were treated with 20% (*w*/*v*) TCA on ice for 5 min, and centrifuged for 15 min at 11,000 RPM. The pellet was suspended in 1 mL of 0.3 % 2-4-ditrophenylyidrazine in 2 M HCl, vortexed and kept in the dark for 1 h, with periodic shaking. Then, 0.5 mL of 50% TCA was added while vortexing the sample, which was kept on ice for 5 min and centrifuged at 11,000 RPM for 5 min. The pellet was suspended in 1 mL ethanol:ethyl acetate (1:1), vortexed and again centrifuged at 11,000 RPM for 5 min. This procedure was repeated three times, and the pellet was later dried with N_2_ gas. After that, 2 mL of 6 M urea were added to the pellet and the sample was incubated at 37 °C for 30 min. The reaction product was measured in a UV-2601spectrophotometer (Beijing Rayleigh Analytical Instrument Corp., Beijing, China) at 370 nm. Each sample was analyzed in triplicate.

### 4.6. Total Protein Measurement

Total proteins were determined by the reaction of tyrosine residues with the Folin-Ciocalteau reagent [76], using bovine serum albumin (BSA) as standard. Briefly, 1 mL of muscle homogenate was mixed with the alkaline copper reagent (0.94 M Na_2_CO_3_, 3.5 mM KNaC_4_H_4_O_6_, 2 mM CuSO_4_, 0.5 M NaOH) and Folin-Ciocalteau reagent (1:20 in distilled water; Merck, Darmstadt, Germany) and incubated at 55 °C for 5 min. Samples were measured at 650 nm in a Rayleigh UV-2601spectrophotometer. Each sample was analyzed in triplicate.

### 4.7. Superoxide Dismutase (SOD) Activity

This assay was performed according Beauchamp and Fridovich [77] that is based on the reduction of cytochrome by the superoxide radical in a xanthine/xanthine oxidase system. Briefly, 5 µL of homogenates from quadriceps or gastrocnemius muscles were mixed with a solution A, composed of 0.5 mM xanthine (Sigma-Aldrich, St. Louis, MO, USA) and 20 µM cytochrome C (Sigma-Aldrich, St. Louis, MO, USA) dissolved in a phosphate buffer (0.1 mM EDTA, 50 mM Na_2_HPO_4_ and 50 mM NaH2PO_4_, pH 7.8) and a solution B containing xanthine oxidase (Sigma-Aldrich, St. Louis, MO, USA) and 0.1 mM EDTA (1:40). Enzymatic activity was detected at 550 nm in a Rayleigh UV-2601spectrophotometer. Each sample was analyzed in triplicate.

### 4.8. Catalase Activity

According to the methods described by Aebi [78], the activity of catalase was determined by spectrophotometrically measuring the loss of absorbance at 240 nm of a reaction mixture consisting of 100 µL of 0.3 M H_2_O_2_ (Merck, Darmstadt, Germany) in 2.9 mL of phosphate buffer (50 mM Na_2_HPO_4_ and 50 mM NaH_2_PO_4_, pH 7.8) and 50 µL of muscle homogenates from quadriceps or gastrocnemius. Measurements were performed during 90 s in a Rayleigh UV-2601 spectrophotometer. Each sample was analyzed in triplicate.

### 4.9. Grip Strength

Kondziela’s inverted screen test was used to measure grip strength of mice using all four limbs [79]. The test was performed by placing the mouse on a square wire screen (43 cm × 43 cm), to later rotate it in 180°. The mouse was suspended upside down above a foam pad and the latency to fall was recorded. The test was performed three times, immediately prior to the onset of the treatment, and at week 8 and at the end (week 10) of the trial. The researchers performing the testing were not blinded.

### 4.10. Statistical Analysis

Sample size was calculated assuming 30% difference in means between control and dysferlin-deficient mice, and considering a power of 90% with a projected *p* value < 0.05 by using an Excel tool from the Illinois Institutional Animal Care and Use Committee (http://iacuc.research.illinois.edu/content/AnimalUse/NumberOfAnimals.aspx). After tests of normality (Kolmogorov–Smirnov), one-way ANOVA followed by Tukey-Kramer multiple comparisons test as post hoc were applied, using 5% significance level. All statistical analyses were performed using GraphPad InStat3 (GraphPad Software Inc, La Jolla, CA, USA). Two-way ANOVA and paired ANOVA for repeated measures were analyzed using IBM SPSS Statistics v24 software (IBM Corporation, Armonk, NY, USA). When Mauchly’s test indicated that the assumption of sphericity had been violated (*χ*22_(2)_ = 13.48, *p* = 0.001), degrees of freedom were corrected using Huynh-Feldt estimates of (ε = 0.80). No outlier test was conducted on the data, and no randomization was performed in this study.

## Supplementary Materials

The following are available online at https://www.mdpi.com/1422-0067/21/12/4293/s1, Table S1: Effects of NAC on oxidative stress parameters and antioxidant enzymes in C57 BL/6 and Bla/J mice, Table S2: Effect of NAC supplementation on muscle strength in C57 BL/6 mice and Bla/J mice.

## Abbreviations

BSA	Bovine serum albumin
MDA	Malondialdehyde
NAC	*N*-Acetylcysteine
Nox2	NADPH Oxidase 2
Nuclear factor κappa B	NF-κB
OS	Oxidative stress
ROS	Reactive oxygen species
SD	Standard deviation
SE	Standard error
SOD	Superoxide dismutase
TAC	Total antioxidant capacity
TBA	Thiobarbituric acid
TBARS	Thiobarbituric acid reactive substances
TEAC	Trolox equivalent antioxidant capacity
TCA	Trichloroacetic acid
Trolox	6-Hydroxy-2,5,7,8-tetramethylchroman-2-carboxylic acid
WT	Wild-type
X-ROS	Nox2-dependent ROS generation
