# Supplementary material for: N-Acetylcysteine Reduces Skeletal Muscles Oxidative Stress and Improves Grip Strength in Dysferlin-Deficient Bla/J Mice
Source: Int J Mol Sci. 2020 Jun 16;21(12):4293. doi: 10.3390/ijms21124293 (PMC7352960; doi:10.3390/ijms21124293)
Supplement: Supplementary file 1 [file ijms-21-04293-s001.pdf]

Table S1. *Effects of NAC on oxidative stress parameters and antioxidant enzymes in C57 BL/6 and Bla/J mice.*

	C57 BL/6 mice	C57 BL/6 mice + NAC	Bla/J mice	Bla/J mice + NAC
Total antioxidant capacity (TEAC, μM)				
Quadriceps	0.86 ± 0.01	0.96 ± 0.004***	0.85 ± 0.005	0.97 ± 0.002&&&
Gastrocnemius	0.86 ± 0.01	0.88 ± 0.005***	0.85 ± 0.005	0.88 ± 0.003&&&
Lipid peroxidation (μmol MDA/mg protein)				
Quadriceps	25.8 ± 11	19.3 ± 9.7	52.8 ± 8.3***	34.8 ± 6.3&
Gastrocnemius	31.6 ± 13	28.4 ± 5.2	45.3 ± 8.8	27.4 ± 5.9&
Protein carbonyl (nmol/mg protein)				
Quadriceps	11.3 ± 3.5	0.5 ± 0.9	69.1 ± 9.2***	39.5 ± 14&&&
Gastrocnemius	9.0 ± 4.8	0.4 ± 0.8*	41.3 ± 6.5***	26.0 ± 6.3&&&
SOD (U/ mg protein)				
Quadriceps	0.16 ± 0.014	0.18 ± 0.003**	0.20 ± 0.012***	0.18 ± 0.002&
Gastrocnemius	0.16 ± 0.004	0.17 ± 0.003	0.18 ± 0.005***	0.17 ± 0.004&&&
Catalase (U/ mg protein)				
Quadriceps	0.07 ± 0.055	0.02 ± 0.004*	0.04 ± 0.014	0.02 ± 0.010
Gastrocnemius	0.03 ± 0.004	0.03 ± 0.005	0.04 ± 0.007*	0.02 ± 0.004&&&

Data are mean ± SD. *p<0.05; **p<0.01; ***p<0.001 compared with C57 BL/6 mice with no NAC treatment. &p<0.05; &&&p<0.001 compared with Bla/J mice with no NAC treatment (one-way ANOVA followed by Tukey-Kramer multiple comparisons test).

Table S2. *Effect of NAC supplementation on muscle strength in C57 BL/6 mice and Bla/J mice*. Muscle strength by using the Kondziela's inverted screen test before and after 1% NAC supplementation for ten weeks.

Mice group	Latency to fall (s) Before treatment (week 0)	Latency to fall (s) Week 8	Latency to fall (s) After treatment (week 10)
C57 BL/6 mice	152 ± 176	142 ± 152	103 ± 66
C57 BL/6 mice + NAC	51 ± 55	48 ± 27	113 ± 131
Bla/J mice	38 ± 48	58 ± 66	39 ± 70
Bla/J mice + NAC	30 ± 16	96 ± 62	149 ± 62

Data show mean ± SD of latency to fall in four groups of animals (C57 BL/6 and Bla/J mice without and with treatment) before and after NAC supplementation. Two-way ANOVA with repeated measures revealed a significant interaction between NAC treatment and measurement time ($F_{(1.60, 31.95)} = 4.53$, $p = 0.025$; $\eta^2 = 0.034$). This interaction was not significant in C57 BL/6 mice ($F_{(1.31, 13,11)} = 1.38$, $p = 0.273$, $\eta^2 = 0.051$), but significant in Bla/J mice ($F_{(2, 20)} = 10.1$, $p = 0.01$, $\eta^2 = 0.129$). A post hoc comparison showed a significant difference at week ten in Bla/J mice ($t_{(15.5)} = -3.32$, p = 0.0004).
